# DENGUE AND CHIKUNGUNYA FEVER: RESURGENT VIRAL INFECTIONS WITH PROMINENT MUCOCUTANEOUS FEATURES

**DOI:** 10.4103/0019-5154.60354

**Published:** 2010

**Authors:** Debabrata Bandyopadhyay

**Affiliations:** *From the Department of Dermatology, Venereology, & Leprosy, R. G. Kar Medical College, 1, Khudiram Bose Sarani, Kolkata 700 004, India.*

Very few clinical situations are as challenging as an acute febrile illness with a rash. The diagnostic possibilities are numerous, ranging from self-limiting to lethal conditions, taxing the acumen of even the most astute clinicians. The most common differential diagnoses include several viral infections, of which two arboviral infections, namely, dengue and chikungunya fever have re-emerged since the beginning of the current millennium as public health problems of global concern.

While dengue is the commonest and the most rapidly spreading mosquito-borne viral disease in the world, chikungunya fever has recently re-emerged after an interval of several decades to affect millions of people, particularly in India. Both the infections have acute fever, joint symptoms, and skin rash as their principal clinical manifestations, are sometimes clinically indistinguishable, and to make the situation worse, may rarely occur as simultaneous co-infections. Laboratory tests remain the cornerstone of accurate diagnosis of both the conditions, but in most epidemic settings these may not be readily available and physicians have to rely on their clinical diagnosis. In this scenario, mucocutaneous features may offer valuable clues to the early diagnosis of these infections.

For both dengue and chikungunya fever, no specific antiviral therapy or vaccines are currently available and the treatment remains symptomatic and supportive. Preventive actions at the personal and community levels in the forms vector control measures and avoidance of mosquito bites are the principal ways of fighting the epidemics. Since the epidemiologic factors responsible for the spread of these infections and the public health inadequacies are likely to persist in the near future, physicians will continue to encounter large number of such cases in the endemic regions of the world. Accurate and updated information on various aspects of these infections could be valuable tools in the hands of clinicians while dealing with these conditions.

I am grateful to the Editor of IJD for allowing me to pull together a series of timely articles for this purpose and to the contributors for their hard work and timely response in sending in the articles.

On offer in this issue's symposium are four articles written by experts on the respective subjects. The masterly article by Gurugama et al. traces the global epidemiologic trends of dengue viral infections and presents a comprehensive overview of the pathogenesis, clinical features, management, and prevention of dengue viral infections. The mucocutaneous features of dengue fever and a detailed discussion on the differentials of the rash have been provided by Thomas *et al*. Readers will find a valuable and elaborate description of the clinical aspects and the epidemiologic features of chikungunya fever with an emphasis on the recent Indian epidemic in the article by Mohan *et al*. Bandyopadhyay and Ghosh have reviewed the mucocutaneous manifestations of chikungunya infection from the available world literature and discussed some unusual features of this re-emerging viral exanthem.

